# The Effect of Intranasal Oxytocin on Social Interaction in Adults and Children With Autism Spectrum Disorders: A Systematic Review and Meta-analysis of Randomized Controlled Trials

**DOI:** 10.31083/AP45322

**Published:** 2026-07-31

**Authors:** Gengjing Fang, Wuyuan Pan, Guokai Li, Le Ma, Jinbao Wei

**Affiliations:** ^1^Department of Pharmacy, Fujian Maternity and Child Health Hospital, College of Clinical Medicine for Obstetrics & Gynecology and Pediatrics, Fujian Medical University, 350001 Fuzhou, Fujian, China; ^2^Department of Pharmacy, The Third Hospital of Xiamen, Fujian University of Traditional Chinese Medicine, 361100 Xiamen, Fujian, China; ^3^Department of Pharmacy, Xiamen Haicang Hospital, 361026 Xiamen, Fujian, China; ^4^Department of Gynaecology Area 2, Fujian Maternity and Child Health Hospital, College of Clinical Medicine for Obstetrics & Gynecology and Pediatrics, Fujian Medical University, 350001 Fuzhou, Fujian, China; ^5^Shanghai Mental Health Center, Shanghai Jiao Tong University School of Medicine, 201108 Shanghai, China

**Keywords:** autism spectrum disorder, oxytocin, social cognition, meta-analysis

## Abstract

**Background:**

Autism spectrum disorder (ASD) is a complex neurodevelopmental condition characterized by marked heterogeneity in cognitive, behavioral, and social functioning. Although oxytocin has been extensively investigated as a potential therapeutic agent for ASD, evidence regarding its clinical efficacy remains inconsistent and inconclusive. This systematic review and meta-analysis aimed to evaluate the therapeutic efficacy of intranasal oxytocin in improving core behavioral and social symptoms in individuals diagnosed with ASD by synthesizing evidence from randomized controlled trials.

**Methods:**

A systematic search of PubMed, Web of Science, and the Cochrane Library was conducted to identify peer-reviewed studies published up to February 16, 2025. Two independent reviewers performed study screening, data extraction, and critical appraisal of methodological quality and strength of evidence. Autism-related behavioral symptom scores were used as outcome measures. Meta-analyses were conducted using random-effects models.

**Results:**

Twelve randomized controlled trials encompassing a total of 733 participants met the inclusion criteria. Pooled results from the random-effects model indicated no statistically significant effect of oxytocin on social functioning outcomes (standardized mean difference [SMD] = −0.05, 95% confidence interval [CI]: −0.20 to 0.10; *p* = 0.54). Between-study heterogeneity was low (I^2^ = 4%; τ^2^ = 0.00). Exploratory subgroup analyses showed no significant differences across pre-specified moderators (all *p* > 0.05).

**Conclusions:**

The current body of evidence does not support a significant therapeutic effect of intranasal oxytocin on core behavioral symptoms in individuals with ASD. However, methodological limitations of the existing literature—including small sample sizes and heterogeneity in study design—limit the strength of conclusions that can be drawn regarding its potential clinical utility.

**Registration:**

The study has been registered on https://www.crd.york.ac.uk/prospero/ (registration number: CRD42024500088; registration link: https://www.crd.york.ac.uk/PROSPERO/view/CRD42024500088).

## Main Points:

1. This meta-analysis of 12 randomized controlled trials (733 participants) found no statistically significant effect of intranasal oxytocin on social functioning outcomes in individuals with autism spectrum disorder (ASD).

2. Exploratory subgroup analyses revealed no significant differences across any pre-specified moderators.

3. The current evidence does not support a significant therapeutic benefit of intranasal oxytocin for improving core behavioral and social symptoms in adults or children with ASD.

4. However, methodological limitations of existing studies—including small sample sizes and heterogeneous designs—warrant caution when interpreting the clinical generalizability of these null findings.

## 1. Introduction

Autism spectrum disorder (ASD) is a neurodevelopmental disorder that is characterized by persistent social interaction impairments, which are associated with restricted and repetitive patterns of behaviors and interests that interfere with quality of life [1]. ASD has been diagnosed in 1% of children worldwide [2]. The male risk for ASD is more than 3.8 times that of females [3]. The increased risk for ASD may also be attributed to factors such as immune dysfunction, maternal vitamin D insufficiency, atherosclerosis, diabetes, obesity, cardiometabolic diseases, preterm delivery, and oxytocin gene polymorphism [4,5,6]. The dysfunctional oxytocin system has long been regarded as one of the primary underlying factors contributing to ASD [7].

Oxytocin (OT) is a neurohormone produced by the supraoptic nucleus (SON) and the paraventricular nucleus (PVN) of the hypothalamus in the brain and is implicated in the regulation of social behaviors, emotions, and functions, including social interactions, social cognition, anxiety-related behaviors, and autonomic-related functions [8,9]. OT is synthesized in the hypothalamus and reaches target regions that express OT receptors (OTRs) and, to some extent, vasopressin V1a receptors “V1aR” [10], both through diffusion and local release of the peptide [11,12]. The effect of OT on human behavior depends on its binding to oxytocin receptors distributed throughout the body [13]. OTRs have been found in the reproductive structure, prefrontal cortex, amygdala, hypothalamus, olfactory nucleus, hippocampus, periaqueductal gray, striatum, nucleus accumbens, ventral tegmental area, lateral septum, medial preoptic area, etc. [14,15]. The oxytocinergic system plays a vital role in shaping social behavior [13]. Oxytocin is expected to be a potential therapeutic resource for the social core symptoms of ASD since this neuropeptide can modulate human social behavior and cognition [16]. Almost all studies exploring the impact of OT on social behavior and cognition have used intranasal delivery [17,18,19]. Nasal delivery provides a means to deliver molecules to the central nervous system (CNS) when other modes of OT delivery exhibit poor bioavailability, and it can bypass the blood-brain barrier (BBB) [20]. Due to its promising therapeutic benefits in enhancing social recognition and related behavioral interventions, intranasal oxytocin administration has been explored as a potential approach to improving neuropsychiatric disorders characterized by impaired social recognition, particularly ASD [21,22].

However, the therapeutic efficacy of OT administration in addressing core symptoms of ASD remains inconclusive across clinical studies [23,24]. While recent randomized controlled trials (RCTs) employing extended intervention protocols (e.g., 6-week treatment combined with active social engagement) demonstrated significant improvements in standardized social responsiveness metrics, including the Autism Diagnostic Observation Schedule, 2nd edition (ADOS-2) and the social responsiveness scale-2 (SRS-2) scores [25], early investigations reported that no treatment-specific improvements in core social symptoms, current observations of long-term beneficial effects particularly on repetitive behaviors and feelings of avoidance are promising and suggest a therapeutic potential of oxytocin treatment for ASD [26]. This discrepancy highlights the critical influence of protocol design variables, particularly the integration of behavioral interventions with pharmacological administration. Future studies should conduct new meta-analyses to examine interactions between neuropeptide administration parameters (e.g., dose, treatment duration) and patient-specific characteristics (e.g., age, sex) on treatment efficacy. Such precision medicine approaches could optimize therapeutic outcomes by identifying patient subgroups most likely to benefit from oxytocin-based neuromodulation while minimizing unnecessary exposure in non-responsive populations.

## 2. Materials and Methods

This systematic review and meta-analysis adhered to the Preferred Reporting Items for Systematic Reviews and Meta-Analyses (PRISMA) guidelines. The study protocol was prospectively registered in PROSPERO on 26 June 2024 (Registration No. CRD42024500088), https://www.crd.york.ac.uk/PROSPERO/view/CRD42024500088.

### 2.1 Search Strategies

We systematically searched PubMed, Web of Science, and the Cochrane Library until February 16, 2025, without language restrictions. The groups of search keywords included were: “Autism Spectrum Disorder” OR “Autism Spectrum Disorders” OR “Autistic Spectrum Disorder” OR “Autistic Spectrum Disorders” OR “ASD” OR “autism” OR “Autistic” AND “Oxytocin” OR “Ocytocin” OR “Syntocinon”. A detailed search strategy is presented in **Supplementary Table 1**.

### 2.2 Study Selection

Studies were selected independently by two researchers and checked to prevent potential errors. A third independent researcher resolved disputes arising during the study selection process. Studies that met the following criteria were included: (1) people with ASD; (2) comparison between the oxytocin group and placebo group; (3) scores for social functioning measured by an ASD scale; (4) RCTs. Studies that met one of the following criteria were excluded: (1) combination therapy or using other medicine, (2) no available data (including studies without reported outcomes, or full-text publications for which outcome data remained inaccessible even after contacting the authors).

### 2.3 Outcome Measures

The primary outcome was social functioning. To be included, studies had to measure this construct using an ASD scale. For this meta-analysis, an “ASD scale” was defined as a standardized and validated instrument specifically designed to assess core social communication and interaction symptoms characteristic of autism. The prespecified eligible scales included the SRS, all versions, the ADOS social-affect and communication subscales, and the Clinical Global Impression-Severity scale (CGI-S), which is used to assess the severity of social impairments globally. Studies that utilized other scales were included only if two independent reviewers judged the instrument to meet the above definitional criteria. The outcome data were extracted as continuous variables based on the scores reported from these scales.

### 2.4 Data Extraction

Data were extracted independently by two researchers and checked to prevent potential errors. A third independent researcher resolved disputes arising during data extraction. From the eligible studies, we extracted the following data: the name of the first author; publication year; the country where the trial was done; details of the trial and patient characteristics (sample size, age range, percentage of males, intervention and control group (participants’ number and its’ dose), intervention duration, follow-up period, scale on autism, dose of OT), scores for autism-related behavioral symptom such as SRS-2, ADOS, CGI-S, etc.

When multiple follow-up time points were reported, data collected at 0-week follow-up (immediately post-intervention) were preferentially extracted. For multi-arm trials, intervention arms were combined into a single group to avoid double-counting of participants, in accordance with the Cochrane Handbook for Systematic Reviews of Interventions.

### 2.5 Study Quality Assessment

In line with the PRISMA guidelines (see **Supplementary Material**), we assessed the risk of bias in the included RCTs using Review Manager (Version 5.3, The Cochrane Collaboration, Copenhagen, Denmark). The evaluation covered seven domains: (1) random sequence generation; (2) allocation concealment; (3) blinding of participants and personnel; (4) blinding of outcome assessment; (5) incomplete outcome data; (6) selective reporting; and (7) other bias. Each domain was categorized as low risk, unclear risk, or high risk.

### 2.6 Data Analysis

All meta-analyses and data visualizations were performed using Review Manager (Version 5.3, The Cochrane Collaboration, Copenhagen, Denmark) or Stata 18.0 (Stata Corporation, College Station, TX, USA). For each included study, data extraction focused on the mean and standard deviation (SD) of score changes on ASD scales from baseline to endpoint for both the intervention and control groups. When the SD of the change score was not directly reported and was unavailable from the study authors, it was imputed using the baseline and endpoint SDs, assuming a correlation coefficient of 0.5 between measurements, as per the Cochrane Handbook recommendations. The standardized mean difference (SMD) with 95% confidence intervals (CIs) was then calculated as the effect size measure using Hedges’ g, which accounts for small sample sizes. We justified the use of a random-effects model for the primary meta-analysis based on the significant clinical diversity (e.g., wide ranges in participant age, intervention dose, and treatment duration) and methodological diversity (e.g., use of different outcome instruments) among the eligible studies. We presumed that the actual effect size would vary across studies. The random-effects model, which incorporates an estimate of the between-study variance (*τ^2^*), was therefore considered more appropriate than a fixed-effect model as it provides a more conservative estimate and allows for generalization beyond the included studies. Heterogeneity was quantified using the *I^2^* statistic and the *τ^2^* statistic. The Egger test was used to assess publication bias.

As pre-specified in our protocol (CRD42024500088), subgroup analyses were planned to assess effect modification by age (children vs. adults), biological sex (male vs. female and male), intervention duration (short-term ≤6 weeks vs. long-term >6 weeks), follow-up duration (0 week vs. >0 week), and oxytocin dosage (low dose ≤24 IU vs. high dose >24 IU). We note that these analyses were underpowered, and their findings are therefore exploratory and hypothesis-generating. Sensitivity analyses were performed to confirm the robustness of the results by removing one study and repeating the meta-analysis. All tests were two-sided, and *p *< 0.05 was considered statistically significant.

## 3. Results

### 3.1 Study Selection and Study Characteristics

**Fig. 1.**
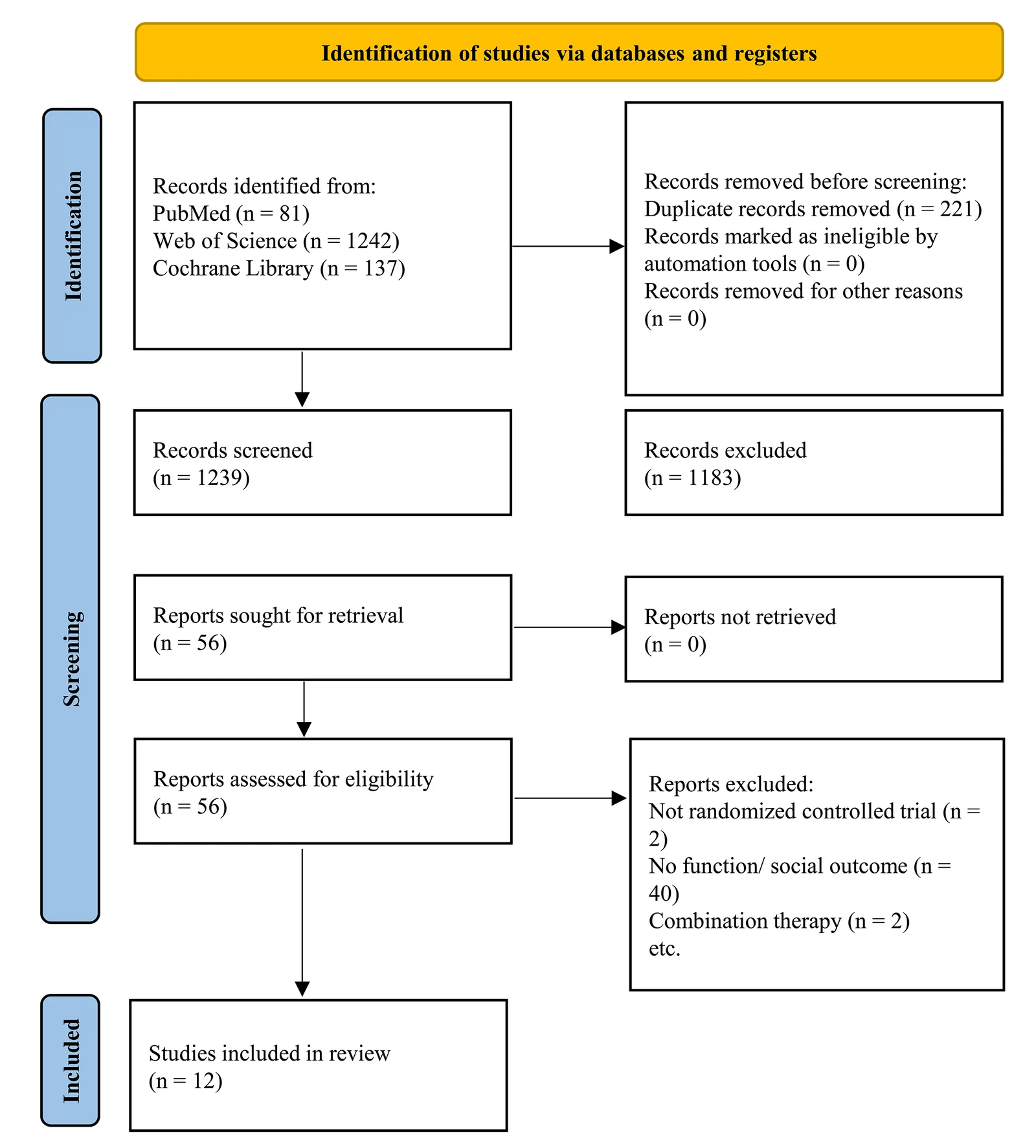
**Flow chart and results of literature screening**.

We identified 1460 articles through searches of PubMed, Web of Science, and Cochrane Library. After removing duplicates, 1239 candidate articles remained. Following title and abstract screening, 1183 articles were excluded. After full-text review, 44 additional articles were excluded. Fig. 1 presents the study selection flowchart and detailed exclusion reasons. Ultimately, 12 articles [25,26,27,28,29,30,31,32,33,34,35,36] were included in this systematic review.

The main characteristics of the included studies are summarized in Tables 1,2 (Ref. [25,26,27,28,29,30,31,32,33,34,35,36]). These studies were published between 2012 and 2023, with sample sizes ranging from 18 to 175 participants (total N = 733). The proportion of male participants ranged from 70% to 100%, and participant ages ranged from 3 to 60 years. All studies administered oxytocin as the intervention, with doses ranging from 3 IU to 48 IU. The intervention duration ranged from 4 days to 12 weeks, and the follow-up period ranged from 0 weeks (immediate post-intervention) to 3 weeks. The outcomes were measured by SRS, ADOS, CGI-S, ADOS reciprocity, SRS-Adult version (SRS-A), SRS-Preschool version (SRS-P), and SRS-2.

**Table 1.** **Basic characteristics of studies included in the systematic review**.

Author (Year)	Country	Age (Years)	Sample size	Intervention group (OT)	Control group (PL)	Intervention duration	Scale on autism
Guastella, A. J (2023) [27]	Australia	3–12	87	32 IU/d, n = 45	n = 42	12 weeks	SRS-2
Daniels, N (2023) [28]	Belgium	8–12	76	24 IU/d, n = 38	n = 38	4 weeks	SRS-2
Yamasue, H (2022) [29]	Japan	18–55	175	3 IU/d, 6 IU/d, 10 IU/d, 20 IU/d, n = 89	n = 86	4 weeks	ADOS reciprocity
Le, J (2022) [25]	China	3–8	41	24 IU/d, n = 21	n = 20	6 weeks	SRS-2
Bernaerts, S (2020) [26]	Belgium	18–35	40	24 IU/d, n = 22	n = 18	4 weeks	SRS-A
Yamasue, H (2020) [36]	Japan	18–48	103	48 IU/d, n = 51	n = 52	6 weeks	ADOS reciprocity
Kosaka, H (2016) [30]	Japan	15–39	55	16 IU/d, 32 IU/d, n = 37	n = 18	12 weeks	CGI-S
Yatawara (2016) [35]	Australia	3–8	31	24 IU/d, n = 15	n = 16	5 weeks	SRS-P
Watanabe, T (2015) [34]	Japan	24–43	18	48 IU/d, n = 9	n = 9	6 weeks	ADOS
Guastella, A. J (2015) [31]	Australia	12–18	50	18 IU/d, 26 IU/d, n = 26	n = 24	8 weeks	SRS
Dadds, M. R (2014) [32]	Australia	7–16	38	12 IU/d, 24 IU/d, n = 19	n = 19	4 days	SRS
Anagnostou, E (2012) [33]	USA	18–60	19	48 IU/d, n = 10	n = 9	6 weeks	SRS

SRS-2, Social Responsiveness Scale-2; ADOS, Autism Diagnostic Observation Schedule; SRS-A, SRS-Adult version; CGI-S, Clinical Global Impression-Severity scale; SRS-P, SRS-Preschool version.

**Table 2.** **Summary of the included Studies’ Characteristics in the meta-analysis**.

Author (Year)	Age	Male (%)	Sample size (Intervention/Control)	Dose of OT	Intervention duration	Follow-up duration	Change in score (Intervention/Control)
Guastella, A. J (2023) [27]	3–12	85.1%	45/42	32 IU/d	12 weeks	3 weeks	–15.68 (25.70)/–16.51 (28.49)
Daniels, N (2023) [28]	8–12	79.2%	38/38	24 IU/d	4 weeks	0 week	–4.08 (10.05)/–4.55 (10.23)
Yamasue, H (2022) [29]	18–55	100%	89/86	3–20 IU/d	4 weeks	0 week	–0.17 (1.51)/0.02 (1.17)
Le, J (2022) [25]	3–8	92.7%	21/20	24 IU/d	6 weeks	0 week	–11.32 (15.14)/–1.16 (10.54)
Bernaerts, S (2020) [26]	18–35	100%	22/18	24 IU/d	4 weeks	0 week	–5.55 (11.40)/–1.06 (10.01)
Yamasue, H (2020) [36]	18–48	100%	51/52	48 IU/d	6 weeks	0 week	–0.80 (1.70)/–1.00 (1.80)
Kosaka, H (2016) [30]	15–39	86%	18/9	16 IU/d	12 weeks	0 week	–0.50 (0.90)/–0.60 (1.05)
Kosaka, H (2016) [30]	15–39	70%	19/9	32 IU/d	12 weeks	0 week	–0.60 (1.14)/–0.60 (1.05)
Kosaka, H (2016) [30]	15–39	100%	37/18	16–32 IU/d	12 weeks	0 week	–0.55 (1.02)/–0.60 (1.05)
Yatawara (2016) [35]	3–8	87%	15/16	24 IU/d	5 weeks	0 week	–10.60 (25.44)/–4.60 (21.93)
Guastella, A. J (2015) [31]	12–18	100%	26/24	18 IU/d (12–15y) 24 IU/d (16–18y)	8 weeks	0 week	–12.07 (22.47)/–12.40 (25.97)
Watanabe, T (2015) [34]	24–43	100%	9/9	48 IU/d	6 weeks	0 week	–6.70 (22.11)/–1.80 (26.15)
Dadds, M. R (2014) [32]	7–16	100%	19/19	12 IU/d (<40 kg) 24 IU/d (>40 kg)	5 days	0 week	1.60 (6.13)/–2.22 (7.96)
Anagnostou, E (2012) [33]	18–60	84.2%	10/9	48 IU/d	6 weeks	0 week	19.10 (25.93)/12.00 (20.22)

### 3.2 Risk of Bias Assessment

**Fig. 2.**
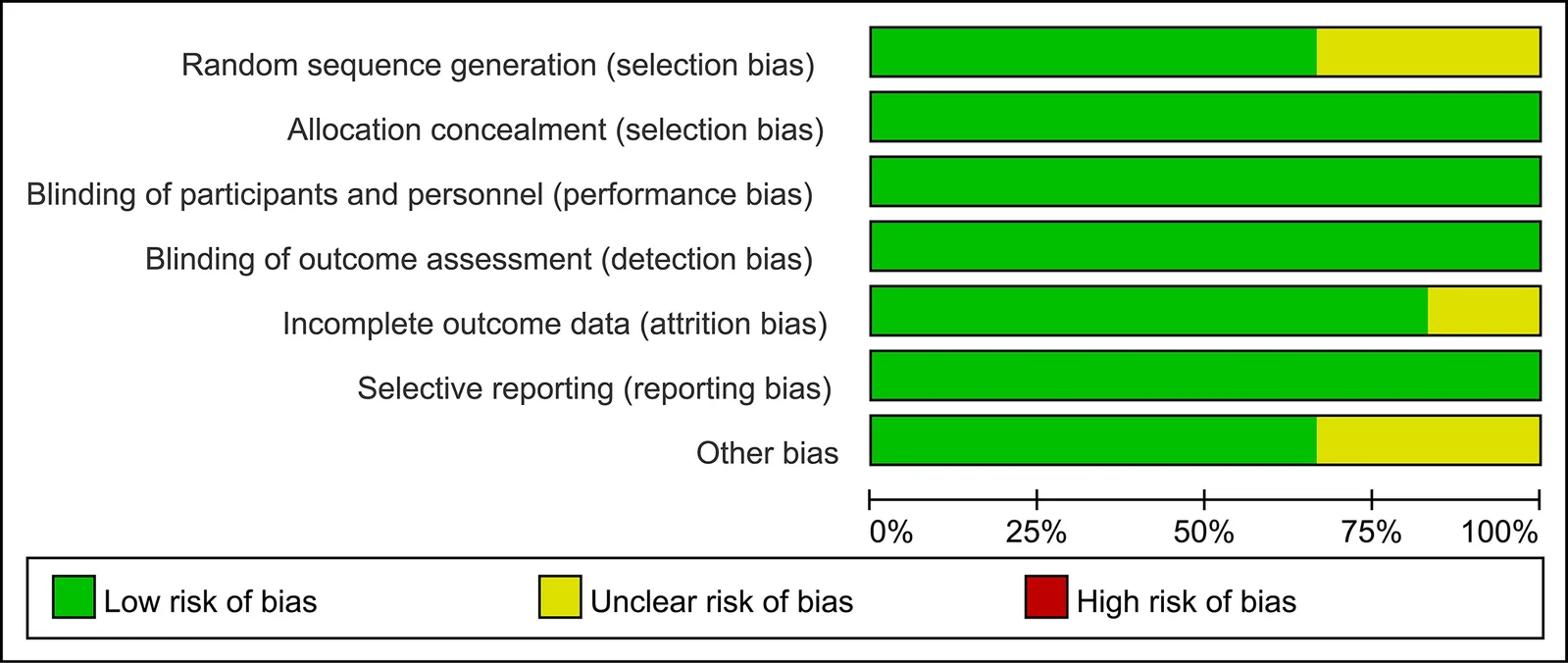
**Risk of bias graph: each risk of bias item presented as percentages across all included studies**.

**Fig. 3.**
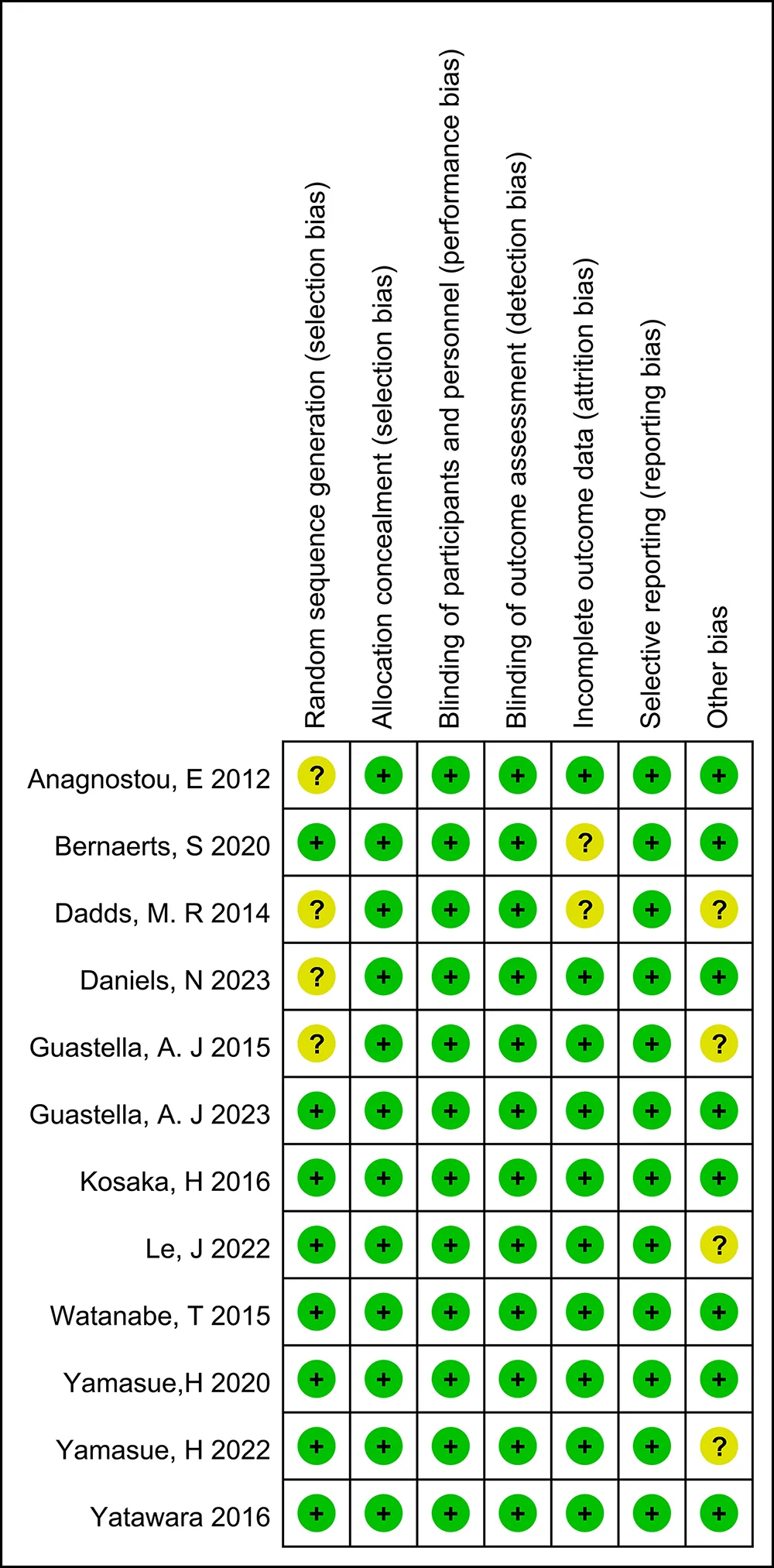
**Risk of bias summary: each risk of bias item for each included study**. Green: low risk of bias; yellow: unclear risk of bias.

All studies were evaluated according to the seven dimensions recommended by the PRISMA guidelines. Among the included studies (n = 12), all demonstrated low risk of bias in allocation concealment (selection bias), performance bias, detection bias, and reporting bias. The majority of studies (n = 10) exhibited low risk of attrition bias. However, several studies rated as unclear risk of bias on random sequence generation (selection bias) and other bias. Among the included studies, five [27,30,34,35,36] met high-quality criteria, while the remaining studies [25,26,28,29,31,32,33] were classified as average quality. These reviewed studies were of average quality overall. Detailed results are shown in Figs. 2,3.

### 3.3 Results of the Meta-Analysis

#### 3.3.1 Efficacy of Oxytocin for Core Symptoms of Autism Spectrum Disorder

**Fig. 4.**
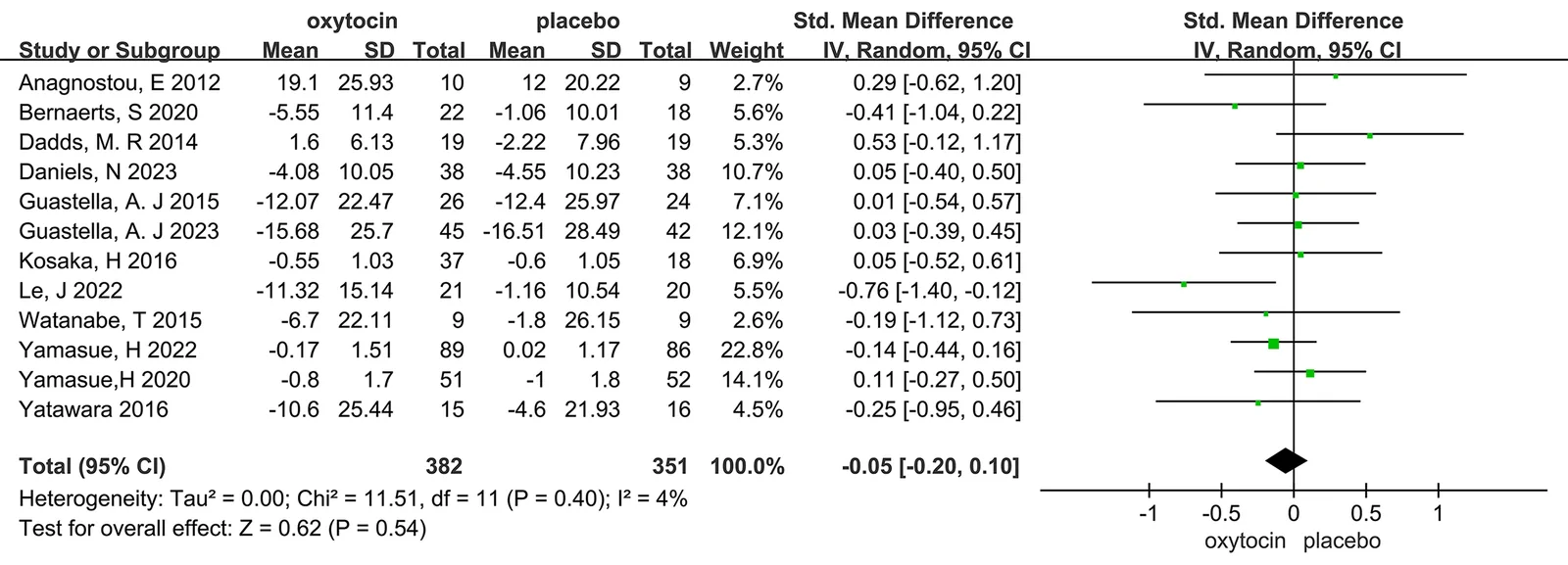
**Forest plot of the overall effect of oxytocin on social functioning in autism spectrum disorder**.

A total of 12 studies published after 2010 were included in the meta-analysis; their characteristics are summarized in Table 2. A random-effects model was employed a priori to provide a conservative, generalizable estimate that accounts for anticipated clinical and methodological diversity. The analysis indicated low between-study heterogeneity (*p* = 0.40, *I^2^* = 4%). The pooled result showed no statistically significant effect of the intervention on autism-related behavioral symptoms (SMD = –0.05, 95% CI: –0.20 to 0.10; Z = 0.62, *p* = 0.54). The forest plot for this analysis is presented in Fig. 4.

#### 3.3.2 Exploratory Subgroup Analyses

**Fig. 5.**
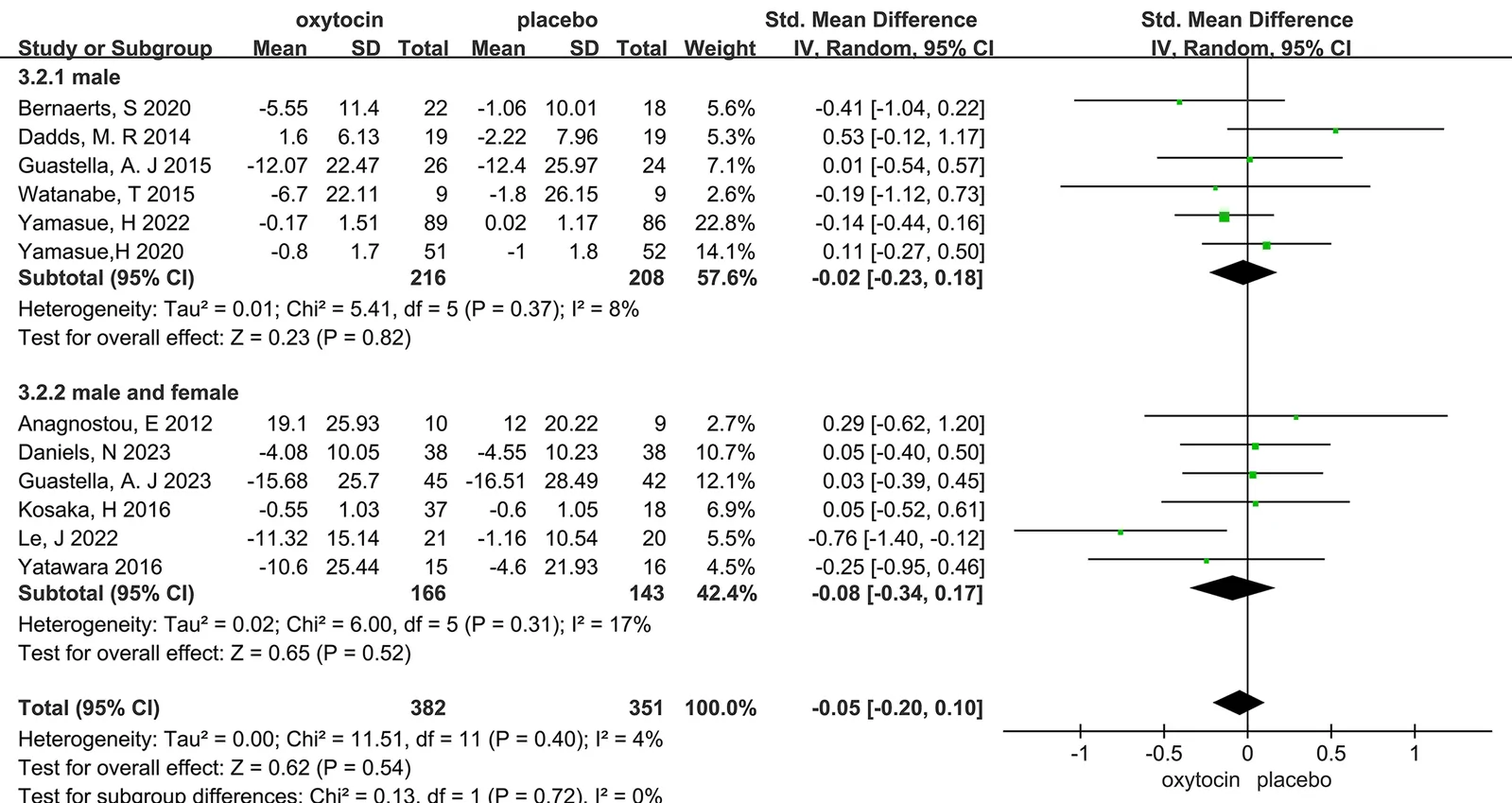
**Forest plot of subgroup analysis by sex**.

**Fig. 6.**
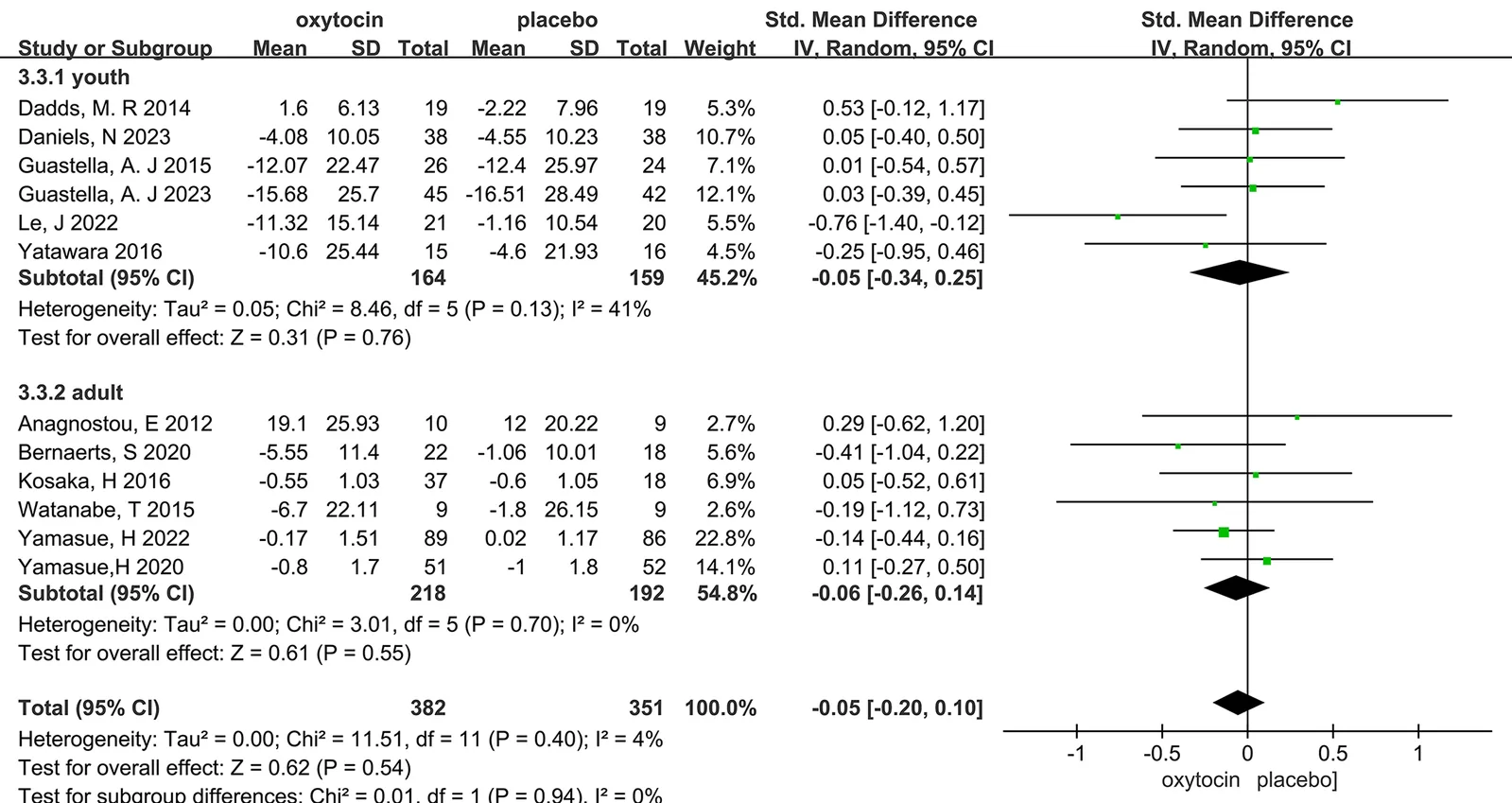
**Forest plot of subgroup analysis by age**.

**Fig. 7.**
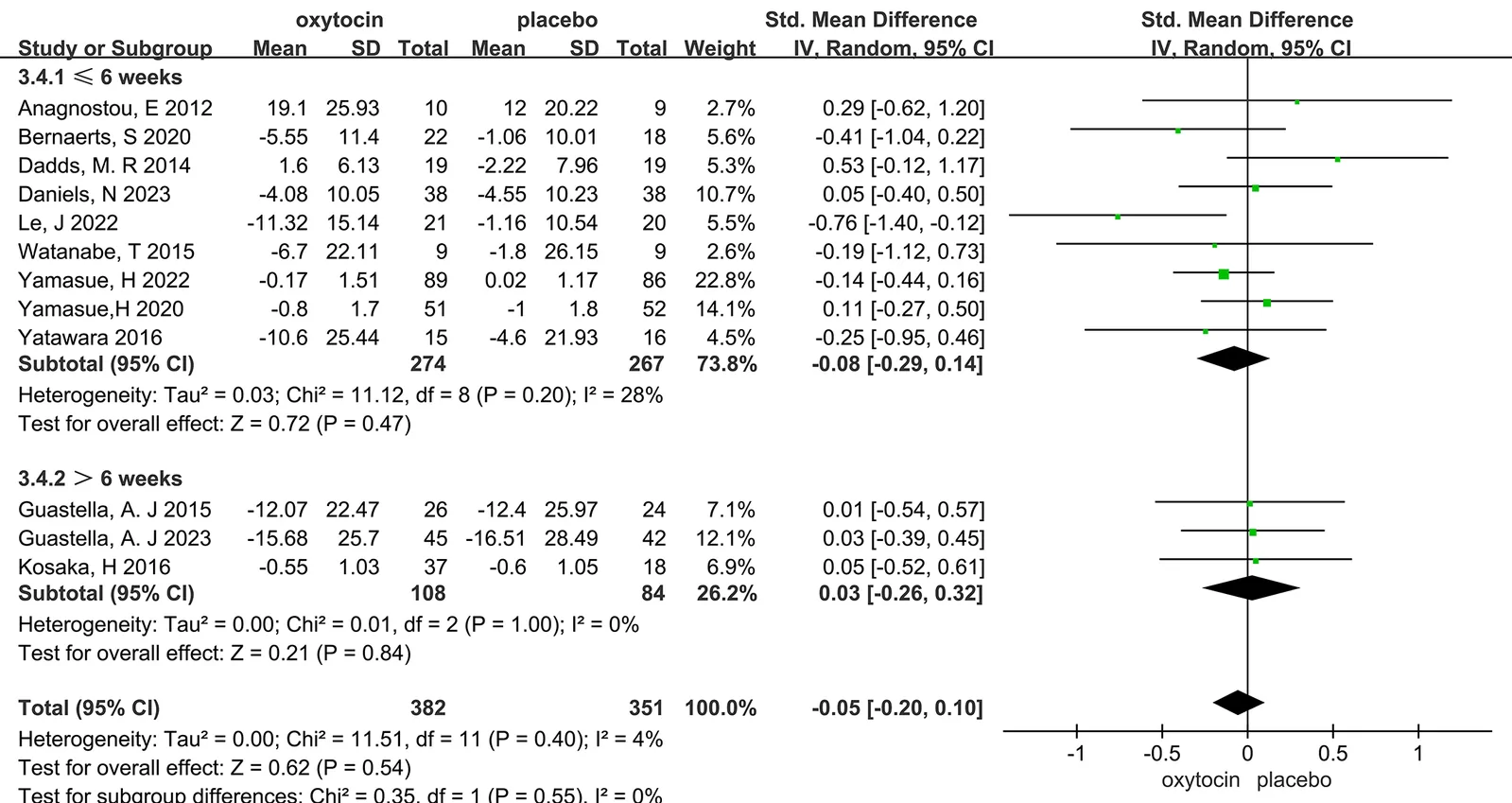
**Forest plot of subgroup analysis by durations of intervention**.

**Fig. 8.**
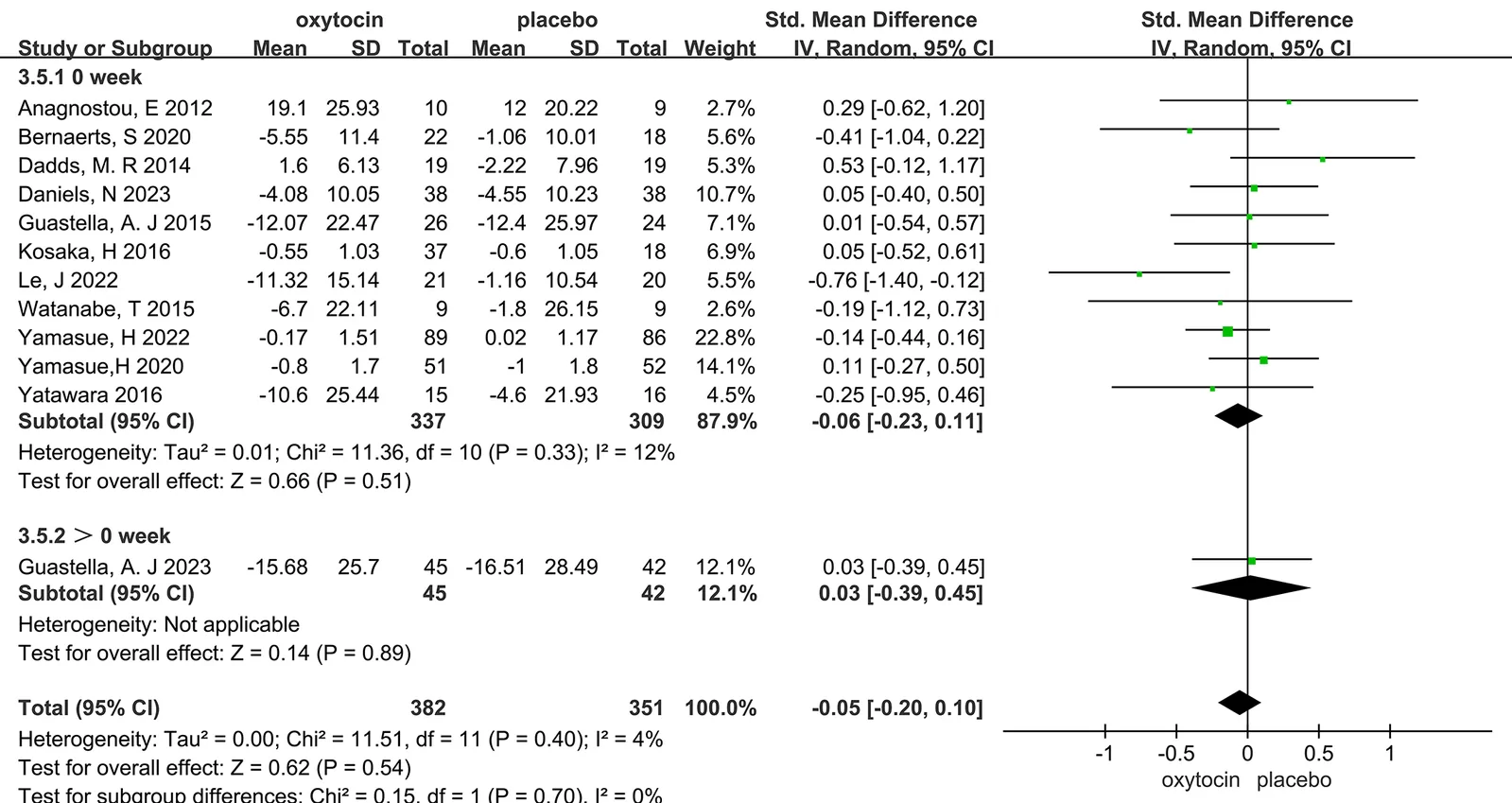
**Forest plot of subgroup analysis by follow-up time**.

**Fig. 9.**
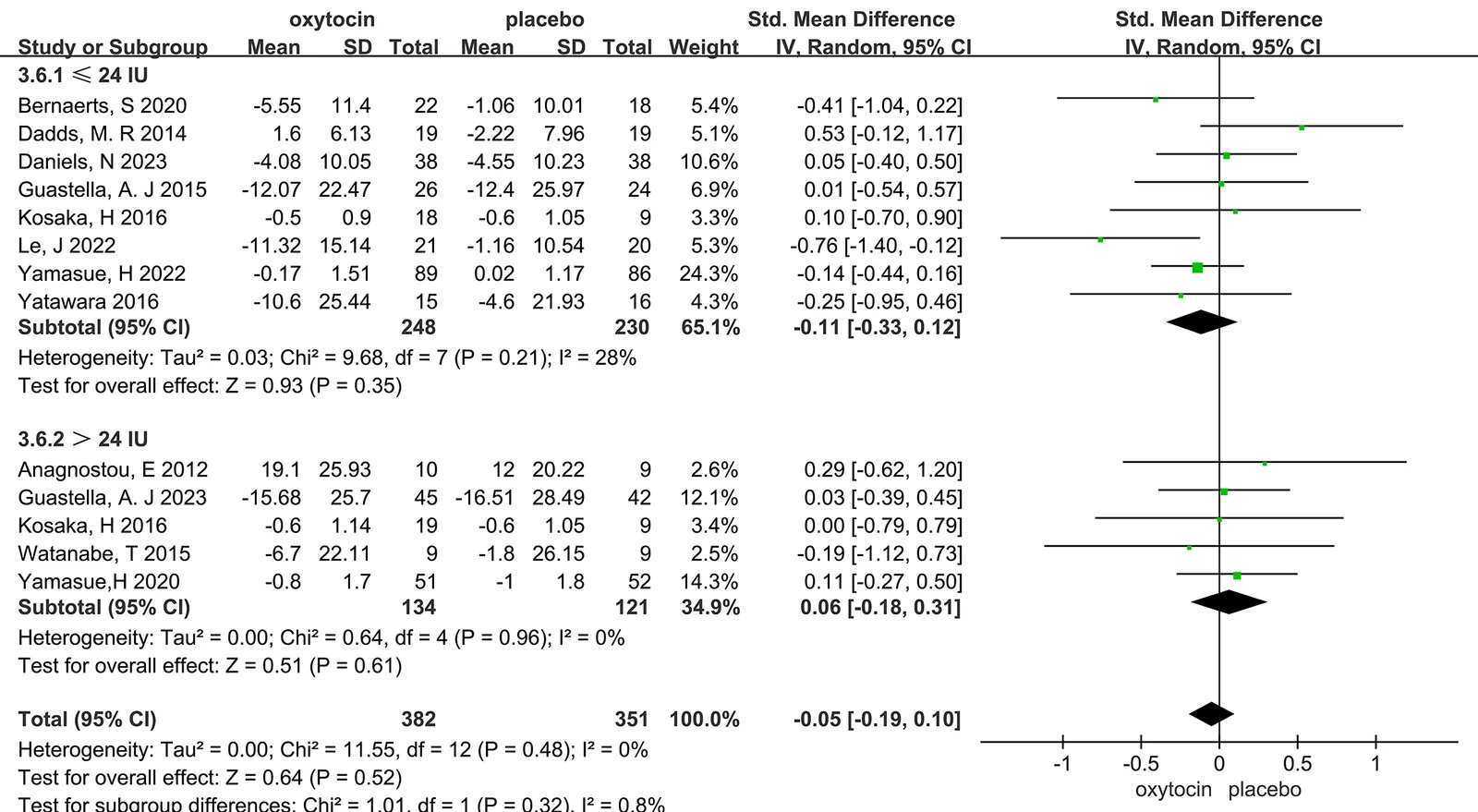
**Forest plot of dose subgroup analysis**.

To explore potential sources of heterogeneity and identify factors that might modify the intervention effect, we performed pre-specified subgroup analyses. The subgroups were defined as follows: sex: male and female; age: youth and adult; intervention duration: ≤6 weeks and >6 weeks; follow-up time: 0 weeks and >0 weeks; dosage: ≤24 IU and >24 IU. The results of these analyses are detailed in Figs. 5,6,7,8,9. Consistent with the non-significant primary outcome, ​the tests for subgroup differences did not reveal any statistically significant moderating effects for any of the variables examined​ (all *p*-values for interaction > 0.05). This indicates that we found no robust evidence that the treatment effect differed across these subgroups.

#### 3.3.3 Publication Bias and Sensitivity Analysis

Twelve studies were included in this meta-analysis. The Egger test (**Supplementary Fig. 1**) was used to assess publication bias, suggesting a low likelihood of publication bias (*p* = 0.8786). Furthermore, a sensitivity analysis was conducted to assess the stability of the meta-analysis results by eliminating studies one at a time. Excluding any single study did not significantly alter the overall effect size, indicating that the findings of this meta-analysis are robust. Moreover, we performed a comprehensive sensitivity analysis to test the robustness of our results to the assumed correlation coefficient (r = 0.5) used for data imputation. We re-ran the primary meta-analysis using a range of values: r = 0.4, 0.5 (primary), 0.6, and 0.8. As presented in Table 3, varying the assumed correlation coefficient from 0.4 to 0.8 resulted in minimal changes in the pooled effect estimate, with SMDs ranging from –0.05 to –0.04. The corresponding confidence intervals remained largely unchanged and consistently included the null value across all scenarios. Although heterogeneity estimates increased modestly with higher assumed correlations—reaching *I^2^* = 32% and *τ^2^* = 0.03 at r = 0.8—the overall findings were stable. These findings demonstrate that our primary estimate is robust and that a correlation coefficient of 0.5 represents a reasonable and conservative choice that minimizes between-study heterogeneity and variance while yielding a stable effect estimate.

**Table 3.** **Sensitivity analysis of assumed correlation coefficients for data imputation**.

Assumed correlation coefficient (r)	Pooled SMD	95% confidence interval	*I^2^*	*τ^2^*	*p*
0.4	–0.05	–0.20 to 0.10	0%	0.00	0.45
0.5 (Primary)	–0.05	–0.20 to 0.10	4%	0.00	0.54
0.6	–0.04	–0.20 to 0.11	11%	0.01	0.58
0.8	–0.04	–0.22 to 0.15	32%	0.03	0.70

SMD, standardized mean difference.

## 4. Discussion

This systematic review provides an updated meta-analysis of randomized controlled trials to investigate the efficacy of oxytocin in ameliorating core symptoms of ASD. We expanded the literature search, included additional new studies, and provided an overview of studies on oxytocin treatment for ASD. However, only 12 eligible studies were included in the systematic review [25,26,27,28,29,30,31,32,33,34,35,36]. The pooled results demonstrated no statistically significant therapeutic effect of oxytocin on overall ASD social functioning. The exploratory subgroup analyses did not reveal statistically significant evidence of effect modification by sex, age, intervention duration, dosage, or follow-up time. This overall null finding must be interpreted within the critical constraint of limited statistical power, as these analyses were inherently underpowered due to the small number of studies contributing to each subgroup. Consequently, the absence of significant interaction effects cannot be equated to the absence of true differential responses across these patient and intervention characteristics.

### 4.1 Comparison to the Previous Meta-Analysis

Our primary finding of a non-significant overall effect is consistent with a recent meta-analysis [37], which also found no conclusive evidence for an effect of oxytocin on autism-related social symptoms. Another meta-analysis [38] also failed to confirm the efficacy of oxytocin in treating adult ASD. This convergence of evidence across independent studies strongly suggests that the overall efficacy of oxytocin for social deficits in ASD is, at best, very limited.

In contrast, our results diverge from those of Yi Huang et al. (2021) [24], who reported a significant benefit. Several methodological differences may explain this discrepancy. First, the positive findings by Yi Huang et al. [24] may be influenced by the inclusion of smaller, single-center studies, which are at higher risk of bias, potentially leading to an overestimation of the effect size. Our analysis, which incorporates more recent and larger RCTs, provides a more conservative, up-to-date estimate. Second, there is a fundamental difference in our statistical approaches: while we used the SMD consistently, Yi Huang et al. [24] pooled results from three different effect size metrics (SMCR, SMD, and raw change scores), which may introduce heterogeneity and affect the comparability of the pooled estimate. Finally, the constructs being measured differed: our analysis focused specifically on behavioral symptoms as measured by core autism scales, whereas their assessment incorporated performance-based tasks and anxiety measures, encompassing a broader, potentially distinct set of domains.

Overall, when contextualized within the existing meta-analytic literature, our null finding reinforces a growing body of evidence suggesting limited overall efficacy of oxytocin for core social deficits in ASD. The discrepancy between our results and those reporting positive effects appears to be methodologically driven, stemming from differences in the rigor of included studies, statistical synthesis methods, and the definition of the outcome construct. The weight of evidence from the most recent and methodologically rigorous studies, including our own, points toward a more modest effect.

### 4.2 Limitations

#### 4.2.1 Limitations in Conceptual Heterogeneity

A notable limitation of this meta-analysis is the conceptual heterogeneity introduced by pooling outcomes from different behavioral scales. The included studies utilized a range of instruments, including the Social Responsiveness Scale (SRS and its versions SRS-2, SRS-A, SRS-P), the Autism Diagnostic Observation Schedule (ADOS, including its reciprocity subscale), and the CGI-S. While all these instruments measure core aspects of autism-related symptomatology, they differ in their specific constructs, reporting sources (e.g., clinician-administered vs. parent-reported), and target populations (e.g., adults vs. preschoolers). Pooling these outcomes, while necessary to synthesize the available evidence, results in an overall effect size that represents a composite of related but distinct constructs. This should be considered when interpreting the generalizability of our findings.

#### 4.2.2 Limitations in Measuring Change in Core ASD Symptoms

An important consideration when interpreting the null findings of this and many other ASD intervention trials pertains to the inherent limitations of the available outcome measures. The majority of included studies relied on instruments such as SRS, which, as a parent-reported measure, is susceptible to expectation bias. Furthermore, gold-standard observational tools like the ADOS, while excellent for diagnostic classification, were not originally designed to be sensitive to detecting subtle changes over time within intervention contexts. Newer instruments, such as the Brief Observation of Social Communication Change (BOSCC), have been developed specifically to address this need for change-sensitivity. The consistent failure of trials to demonstrate significant effects on core symptoms may therefore reflect, at least in part, a fundamental mismatch between the interventions being tested and the tools used to measure their outcomes. This underscores a critical challenge for the field and highlights the imperative for future research to prioritize the development and application of more objective, precise, and change-sensitive outcome measures, including potential biomarkers.

#### 4.2.3 Limitations in Sample and Intervention Protocols

The analysis could not assess efficacy in females because no female-only studies were available. Furthermore, the restricted ranges in dosage (primarily 24 or 48 IU/day) and treatment duration (mainly 6 or 8 weeks) among the included trials prevented a robust evaluation of dose- or time-dependent effects. These constraints mean that the non-significant overall effect may partly stem from the failure to identify a more effective, specific treatment protocol rather than a definitive absence of any therapeutic benefit.

Consequently, future research must address two interrelated imperatives: the development of more sensitive, objective outcome measures (including biomarkers) and the conduct of large-scale, preregistered trials with sufficient power to detect subgroup effects. This dual approach is essential to definitively evaluate the potential of oxytocin in treating ASD.

Additionally, recent findings, including those of Le et al. (2022) [25] suggest that oxytocin administration may exert more consistent clinical benefits when paired with structured social interaction or behavioral engagement. Future trials may consider incorporating parent-mediated social activities, therapist-guided social skills training, or other enriched social interaction paradigms in combination with oxytocin. Such combined designs may allow researchers to better evaluate potential synergistic effects and clarify whether oxytocin enhances the salience of social cues primarily in enriched social contexts.

## 5. Conclusions

In summary, this meta-analysis indicates that exogenous oxytocin administration does not yield statistically significant improvements in the core symptoms of ASD. This finding is consistent with previous systematic reviews [38], which also reported limited overall efficacy of oxytocin pharmacotherapy for ameliorating global ASD symptoms. It is important to note, however, that the current body of evidence is constrained by methodological limitations, including small sample sizes, heterogeneous study designs, and variable risk of bias across the included RCTs. Therefore, the clinical generalizability of these non-significant findings should be interpreted with caution.

## Data Availability

All data reported in this paper will be shared by the corresponding author upon reasonable request.

## Abbreviations

ASD, autism spectrum disorder; OT, oxytocin; SON, supraoptic nucleus; PVN, paraventricular nucleus; OTRs, oxytocin receptors; CNS, central nervous system; BBB, blood-brain barrier; RCTs, randomized controlled trials; ADOS-2, Autism Diagnostic Observation Schedule, 2nd edition; SRS-2, Social Responsiveness Scale-2; SRS, Social Responsiveness Scale; CGI-S, Clinical Global Impression-Severity scale; SD, standard deviation; SMD, standardized mean difference; CIs, confidence intervals.
